# School Achievement in Early Adolescence Is Associated With Students’ Self-Perceived Executive Functions

**DOI:** 10.3389/fpsyg.2021.734576

**Published:** 2022-03-14

**Authors:** M. A. J. van Tetering, J. Jolles, W. van der Elst, D. D. Jolles

**Affiliations:** ^1^Faculty of Social and Behavioural Sciences, Educational Sciences, Institute of Education and Child Studies, Leiden University, Leiden, Netherlands; ^2^Denkkracht, Centre for Neuropsychological Expertise, Nijmegen, Netherlands; ^3^Faculty of Behavioral and Movement Sciences, Vrije Universiteit Amsterdam, Amsterdam, Netherlands; ^4^Statistics and Decision Sciences, Johnson & Johnson, Beerse, Belgium

**Keywords:** executive functions (EF), self-report, early adolescence, reading, mathematics, parental education, sex, school achievement

## Abstract

The primary aim of this study was to investigate the relation between self-perceived executive functions (EFs) and the school achievement of young adolescents (aged 10–12 years), while controlling for parental education and sex. We specifically focused on executive aspects of daily life behavior and the higher-order EFs, as measured with self-report, rather than on the more basic EFs which have been the primary focus of prior investigations. In two independent samples of sixth graders (*N* > 200 each), students evaluated their EFs on a self-report questionnaire, the Amsterdam Executive Functioning Inventory. School achievement in the domains of mathematics and reading comprehension were evaluated with nationally used, norm-based achievement tests. Results revealed that the self-perceived EFs of young adolescents were significantly correlated with their school achievement in both study samples. School achievement was also correlated with the level of parental education, but the factor sex did not have such influence. In study 1, self-perceived EFs explained additional variance in school achievement, while controlling for parental education and sex. In study 2, this was only the case for the most robust measure of school achievement, i.e., the end-of-primary-school final achievement test. Furthermore, besides the relation with achievement tests, we also found a relation between self-perceived EFs and teacher ratings behavioral problems in the classroom. Together, our findings imply that young students can properly reflect on the effectiveness and appropriateness of their EFs in a way that is relevant to their academic achievement and classroom behavior. The findings underscore the importance of considering the development of EFs and parental education in the evaluation of academic achievements in early adolescence.

## Introduction

EFs play a crucial role in the academic performance of young adolescents (Benson et al., 2013; Zelazo et al., 2016). This is shown by a substantial number of studies that focus on core EFs—including working memory, inhibition, and cognitive flexibility—and their importance to academic performance (e.g., mathematics and reading ability) during the school years (for an overview, see e.g., Yeniad et al., 2013; Jacob and Parkinson, 2015; Zelazo et al., 2016; Follmer, 2018). In addition to these core EFs, complex daily life situations such as academic learning and classroom behavior rely on several higher-order EFs. For instance, students need to organize their schoolwork and plan activities for the short and long term. They also need to be able to focus on a specific topic for a prolonged period, and to prioritize between activities. This includes prioritizing between different learning strategies, but also between scholastic tasks and competing activities such as leisure, gaming, and sports. Furthermore, students need to be able to select and evaluate the possible consequences of their choices, and reflect on the optimal route to attain goals, and to be creative in problem solving. In the present study, we focus on these higher-order EFs that—in addition to the core EFs—fall under the umbrella of the EFs. Important higher-order EFs which receive much research interest are the regulation of attention in daily life situations, self-control and performance monitoring, planning and prioritizing, and overseeing the consequences of one’s actions (see Lezak, 2004; McCloskey and Perkins, 2012; Diamond, 2013, 2016; and earlier papers of our research group, e.g., van der Elst et al., 2012b; Baars et al., 2015; van Tetering et al., 2018). In the present paper, we investigate the relation between these executive aspects of daily life behavior and the school achievements of young adolescents (10–12-year-old). In contrast to many earlier papers on the relation between EFs and school performance, we focus upon the EFs as perceived by students themselves.

The choice for student self-reports adds a new dimension to the study of EFs in relation to learning performance. Up till now, there has been quite some research that focused upon the core EFs which are usually measured with performance on cognitive tasks (e.g., Butterfuss and Kendeou, 2018). Yet, cognitive tests are not suitable for the evaluation of the higher-order EFs in daily life situations. To evaluate higher-order EFs, observer- or self-reports are the instrument of choice (Lezak, 2004). In most of the earlier studies that focused upon higher-order EFs, it was investigated how teachers or parents evaluated the EFs of the adolescent on an observation scale or questionnaire (e.g., Yeniad et al., 2013; Zelazo et al., 2016; Follmer, 2018). An example of a study in which observer reports were used is that of van Tetering et al. (2018). In this study, teachers evaluated the higher-order EFs of 9–12-year-old students. Teachers rated Students’ EFs on a standardized questionnaire that has been used in several studies in adolescents: the Amsterdam Executive Functioning inventory (AEFI; van der Elst et al., 2012b; Baars et al., 2015; van Tetering et al., 2018, 2020). It was then investigated whether these teacher-evaluations were related to the school achievements of the students. In line with other studies in this domain (e.g., see the studies described by Zelazo et al., 2016; Duckworth et al., 2019), results of van Tetering et al. (2018) showed that young adolescents who were characterized by lower teacher evaluated EFs had poorer academic outcomes than students with higher evaluations. With respect to the observer reports of teachers, it should be noted that they can be influenced by student characteristics such as sex, ethnicity, and social background. This is reported by Garcia et al. (2019). To prevent for this bias, we planned the present study in which we did not use observer-reports but self-reports (self-evaluations) in roughly the same age-group as in van Tetering et al. (2018).

Another advantage of using Students’ self-reports over using parent- or teacher evaluations is that they provide more information on the EFs of students across different day-to-day situations. Parents and teachers do not observe children in all situations, and the intensity of their supervision gradually declines during childhood and adolescence. Accordingly, students likely have a better view of their own EFs in relation to the organization of their schoolwork and their functioning in other daily activities, such as their ability to sustain attention while doing homework and to ignore tempting distractions like gaming, watching television, or meeting up with peers. Self-reports could thus yield relevant information that cannot be obtained by observer report. A practical advantage of the use of self-reports is that they are easy to administer in a classroom setting: it is easier to organize than an observational study in which a teacher is asked to evaluate each individual student. The self-reports of young adolescents may thus be an easier and a more direct method to gain information on the EFs of young adolescents compared to parent- or teacher evaluations.

It is of importance to note that young adolescents are in the process of growth and development and their brain is in maturation up until well in the third decade of life (e.g., Gogtay et al., 2004; Blakemore, 2012; Steinberg, 2014; Zelazo et al., 2016). Accordingly, their self-evaluation/self-insight and self-regulation are in development as well (Zelazo et al., 2008; Diamond, 2013; Steinberg, 2014). Consequently, young teens may overestimate themselves, but the reverse may also be true: quite some young adolescents underestimate their performance and potency as is described by Steinberg (2014); Jury et al. (2015), and Martens and Jolles (2015). Therefore, it is not yet known whether young adolescents are able to provide a valid self-evaluation. With our study, we aim to gain insight into Students’ perceptions of their own abilities by asking them to reflect on their own attentional skills, their planning abilities, and their self-control and self-monitoring skills, and relating these measures to their academic achievement.

As mentioned above, not many studies have examined the relationship between self-reported higher-order EFs and the academic achievements of young adolescents. Yet, some conclusions can be drawn from our prior study involving students aged 12–18 (Boschloo et al., 2014). In this study, the Behavior Rating Inventory of Executive Function Self-Report (BRIEF-SR; Gioia et al., 2000) was used to evaluate EFs in students in the pre-university track of secondary school (Boschloo et al., 2014). Results showed that Students’ evaluations were significantly correlated to their report marks (*r* = 0.17–0.27). Yet, these correlations did not remain significant after controlling for the background characteristics grade, sex, and level of parental education (LPE; Boschloo et al., 2014). More generally, studies that controlled for Students’ background characteristics reported lower correlations between EFs (assessed by behavioral tasks) and academic achievements (around 0.10–0.25, see Latzman et al., 2010; Miller and Hinshaw, 2010) than studies that did not control for background variables (corrections between 0.30 and 0.50 or higher, e.g., St Clair-Thompson and Gathercole, 2006; Best et al., 2011). Together, these findings suggest that the background characteristics grade, sex and LPE may play an important role in the relation between EFs and academic performance. Therefore, we included sex and LPE in our analyses to examine whether the relation between self-reported EFs and academic achievement holds when controlling for these factors.

With respect to the possible influence of LPE, there are strong indications that higher educated parents have more cognitive and financial resources to support their children’s intellectual development than lower educated parents (Rindermann and Baumeister, 2015). For example, higher educated parents generally provide better toys and use more complex and diverse language in interacting with their children (Rindermann and Baumeister, 2015). Furthermore, higher educated parents generally have more financial resources to support their children by providing access to private education or homework classes. These differences in the learning environment provided by higher vs. lower educated parents may contribute to individual variation in children’s cognitive development and their academic achievements. This notion is supported by findings from earlier studies showing a relation between socioeconomic status (SES), including LPE, and children’s EFs (Mezzacappa, 2004; Ardila et al., 2005; Hurks et al., 2006; Moffitt et al., 2011; Hackman et al., 2015). Moreover, EFs have been found to (partially) mediate the relationship between SES and school achievement, suggesting that EFs serve as a potential mechanism underlying the achievement gap between lower and higher SES children (Lawson and Farah, 2017). In line with these findings, we recently showed that teachers reported better EFs in children from higher LPE families than in children from lower LPE families (van Tetering et al., 2018). This finding supports the notion that LPE is a student-related factor that contributes to individual variations in children’s EFs, as expressed in the classroom. Yet, it is important to keep in mind that teacher-evaluations may be biased by their prior knowledge about the sex, language ability and family background of a student (Garcia et al., 2019; Inspectie van het Onderwijs, 2020). Accordingly, teachers may evaluate the abilities of students of higher LPE families more positive in general, indicating that their evaluations might not give an adequate estimation of a Students’ EFs. This bias is prevented by studying the self-perceptions of young students, as we do in the present paper.

In addition to LPE, we investigate whether male or female sex is a child-related factor that contributes to individual differences in the self-perceived EFs of young adolescents. Some earlier studies in which behavioral tasks or observer-reports were used to assess EFs in young adolescents imply that there are sex differences in favor of girls. For instance, behavioral studies have revealed that girls outperform boys on verbal fluency (Barel and Tzischinsky, 2018), information processing speed (van der Elst et al., 2012a), and inhibitory control tasks (Loyer Carbonneau et al., 2020). Furthermore, we have found sex differences in teacher evaluations of EFs in 9–12-year-olds, particularly in the domain of self-control and self-monitoring (van Tetering and Jolles, 2017). These findings imply that the sex of the student may be a factor that contributes to individual differences in the of EFs of young adolescents. Moreover, there appear to be substantial differences in the school performance of boys and girls in early adolescence (OECD, 2015), and it is not unlikely that sex differences in EFs may contribute to these performance differences. This notion is substantiated by a growing literature which shows that there is a correlation between EFs and school performance (e.g., Fuchs et al., 2003; Siegler and Pyke, 2013; Vukovic et al., 2014; Zelazo et al., 2016; Duckworth et al., 2019). If young adolescent girls have stronger EFs than boys, they may be better able to organize their schoolwork and to pay attention during classroom instructions than boys. This may be directly beneficial for their school performance (Duckworth and Seligman, 2006). Nevertheless, it is not clear whether sex differences will be evident in self-perceived EFs of early adolescents. In fact, one of our earlier studies showed sex differences in the self-perceived EFs of 13–16-year-olds, but not in the evaluations of 10–12-year-olds (van Tetering et al., 2020). This finding highlights the importance for further research on sex-differences in the self-perceived EFs of young adolescents, as we do in the current study.

In summary, the primary aim of this study was to investigate the relation between self-perceived EFs and academic performance in early adolescence. Based on findings of earlier work described above, we hypothesize that higher self-perceived EFs are associated with higher academic performance. In addition, we expect LPE and sex to be related to both academic performance and self-perceived EFs as well. Therefore, it is important to investigate whether the relation between self-perceived EFs and academic performance holds when controlling for LPE and sex. Based on prior findings in secondary school children, we expect that the relation between self-perceived EFs and academic performance may reduce or even disappear when controlling for LPE and sex (e.g., Boschloo et al., 2014). These hypotheses were investigated in two different samples, each including more than 200 sixth grade participants, aged 10–12-year. Data were collected by means of group administration in the class environment. In both studies, the AEFI was used to measure self-perceived EFs (van der Elst et al., 2012b; Baars et al., 2015; van Tetering et al., 2018, 2020), and school performance was assessed using standardized achievement tests in two specific cognitive domains: mathematics and reading comprehension. The reason that we included two samples with nearly identical outcome measures was because of the so-called “replication crisis.” This crisis revealed that results of many scientific studies are difficult to replicate or reproduce, especially in the social sciences (Ioannidis, 2005). Yet, in addition to replicating analyses from study 1, study 2 also investigated the relation between self-perceived EFs and performance on the end-of-primary-school final achievement test. This is an important additional outcome measure as this test is used in most primary schools in the Netherlands to assist in the selection of secondary education.

In addition to the primary aim, study 2 addressed two follow-up questions regarding the relationship between self-reported EFs and Students’ functioning in school. In study 2, teachers were asked to answer several questions about their students, two of which were especially relevant in the context of the current investigation. The first question pertains to teachers’ perceptions of Students’ achievement, *considering their academic potential* (Jury et al., 2015; Martens and Jolles, 2015). In other words, rather than asking about Students’ school performance *per se*, which would likely correspond highly with Students’ achievement test scores, teachers were asked to which extent they would characterize each student as an “underachiever” (on a 5-point scale). We expected that students reporting poorer EFs would more often be characterized as underachievers by their teachers, and that this effect holds after controlling for performance on the standardized achievement tests. Second, we were interested in teachers’ perceptions of Students’ classroom behavior. It is increasingly recognized that success in school is more than just obtaining high grades (e.g., Biesta, 2020). Instead, education is thought to be of major importance in guiding children to become independent and well-functioning members of our society. In this context, it is of interest to examine Students’ behavior in the classroom, and the extent to which this is related to their self-reported EFs. As there is an extensive literature relating poor EFs (specifically self-control) to externalizing behavior and delinquency (e.g., Fergusson et al., 2013; Fine et al., 2016), we expected that students with poor EFs would also display disrupting behavior in the classroom, as experienced by the teacher. To study this question, teachers were asked to indicate (also on a 5-point scale) whether each of their students displayed behavioral problems in the classroom including disinhibited, angry, and aggressive behavior.

## Materials and Methods

### Participants

A total of *N* = 232 sixth grade students took part in study 1 (age: *M* = 11.6, *SD* = 0.44, sex: 57.3% girls). In study 2, a total of *N* = 237 sixth grade students were included (age: *M* = 11.8, *SD* = 0.45, sex: 50.2% girls). The participants in study 2 were part of a larger dataset including *N* = 1,081 subjects in grades 2–6 (van Tetering et al., 2019).

Recruitment procedures were similar between the two studies, but there are two differences worth mentioning. First, in study 1, the disclosure of national school achievement test scores was optional, and caregivers gave separate permission for this. In study 2, national school achievement test scores were obtained for all participating individuals and no separate permission was asked. Second, in study 1, participants with learning- or developmental disorders were excluded from participation, whereas in study 2, all students in sixth grade were eligible to participate.

In both studies, it was explained to the parents and students that participation was voluntary, that personal information would be pseudonymized, and that all data would be assembled and analyzed on a group level and thus could not be traced to individuals. The participants and caregivers gave written permission to participate. The Ethics Committee of the Faculty of Educational Sciences of the Leiden University approved the study protocol of study 1, and the Ethical Committee of the Faculty of Behavioral and Movement Sciences of the Vrije Universiteit Amsterdam approved the study protocol of study 2.

### Procedure

In study 1, data were obtained on 12 mainstream primary schools, and in study 2 (i.e., the BrainSquare study, van Tetering et al., 2019), data were obtained on 9 mainstream primary schools. The primary schools of study 1 and 2 were all located in the Western part of the Netherlands in the Amsterdam—Den Haag region, in both cities and rural areas. The data collection procedures were identical in the two studies. There were some differences in the achievement and LPE outcome measures, as reported below. All collaborating schools agreed to include the testing procedure into their regular school schedule.

Data were collected by means of group administration in the classroom environment. One of the researchers gave instructions to the participants and kept track of time. The other walked around in the classroom to assist the participants with procedural problems. A maximum of 33 children were tested together in the classroom. Participants sat at their own table and all tables were separated from each other (approximately 1 m in between). Various questionnaires and neuropsychological tests (not reported here) were administered. Total administration time was approximately 30 min in study 1, and 60 min in study 2.

### Materials

#### Mathematics and Reading Comprehension Performance

In both studies, school performance was assessed by two standardized and nationally norm-referenced paper-and-pencil achievement tests. These tests have been developed by the Dutch Standard Central Institute for Test Development [i.e., in Dutch: Centraal Instituut voor Toets Ontwikkeling (CITO)]. The tests that were used in the present study were the Mathematics and Reading Comprehension tests (for CITO Mathematics see Hop et al., 2019; for CITO Reading Comprehension see Tomesen et al., 2018). These tests are administered annually on most primary schools in the Netherlands to monitor the school performance of individual students across the school years. Yearly, the tests are administered in January and June. In the present paper, results obtained closest to the administration of the executive functioning inventory were used (i.e., June 2019 in study 1 and January 2014 in study 2).

The achievement levels or skills scores from the CITO Mathematics and Reading Comprehension tests were used, which allow for comparison with peers and monitoring of an individual’s progress respectively. For study 1, achievement levels were used as a measure of individual differences in school achievement. Achievement levels are provided on a 5-point scale. Each level represents 20% of the students in a particular grade in the national sample, with 1 representing the highest 20% of students, and 5 representing the lowest 20%. For study 2, achievement levels were not available. Therefore skill scores were used instead. Skill scores provide a measure of the absolute performance of an individual across the school years. They are typically used for monitoring the progression in the domains of mathematics and reading comprehension (de Wijs et al., 2010; Feenstra et al., 2010; Janssen et al., 2010; Mols and Kamphuis, 2012).

The validity and reliability of earlier versions of the tests have extensively been investigated. Reliability coefficient of the CITO Mathematics in sixth grade was 0.95 and its test-retest reliability ranges between 0.95 and 0.96 (Hop et al., 2019). For the CITO Reading Comprehension, reliability coefficient and test-retest reliability ranges from 0.88 to 0.89 in sixth grade (Tomesen et al., 2018). Reliability of both tests can thus be considered to be good. The same applies to their validity. This is because (1) calibration research showed that performance on the three CITO tests could be explained by one unidimensional concept, (2) both CITO test performance scores were highly correlated with similar abilities measured with other parts of the tests, and (3) performance on a particular CITO test was predictive for performance on the following CITO test of that domain (Tomesen et al., 2018; Hop et al., 2019).

##### Mathematics

The Dutch standardized CITO Mathematics test takes 45 min to administer (Hop et al., 2019). Participants fill out their answers on a piece of paper. The following math skills are covered in the test: (a) number and number relations; (b) addition and subtraction, multiplication, and division; (c) measuring (e.g., weights, length, surface, time); (d) percentages and fractions; and (e) understanding tables and graphs.

##### Reading Comprehension

The Dutch standard CITO Reading Comprehension test takes approximately 40 min to administer (Tomesen et al., 2018). The following Reading Comprehension skills are covered: (a) understanding written texts, (b) interpreting written tests, (c) summarizing written texts, (d) finding information in written texts.

##### CITO Final Achievement Test

In study 2, school performance was additionally assessed using a standardized and nationally norm-referenced paper-and-pencil end-of-primary-school achievement test, i.e., the CITO Final Achievement Test. This test is yearly administered (in May) in sixth grade by most Dutch primary schools. The CITO Final Achievement Test has been developed to aid in the process of choosing secondary education. Data on the CITO Final Achievement Test consist of 165 multiple-choice questions in the areas of language and arithmetic-mathematics. Performance is provided on a scale of 501–550 (van der Wal, 2020); higher scores indicate better performance.

#### Level of Parental Education

In study 1, LPE was rated on a classification system that is based on the International Standard Classification of Education (Singh, 2010). This is a statistical framework on maintained education that is suitable for assembling, compiling, and presenting education statistics, both within individual countries and internationally. The rating scale used in this study was adjusted according to the Dutch educational system. Accordingly, caregivers indicated their highest level of education in their family on a 7-point scale (0 = no finished education to 6 = post university). Because of the skewed distribution of this variable in our population, LPE was divided into three categories (low ≤ 4, moderate = 5 or 6, and high = 7). In study 2, a self-report measure of LPE was not available. Instead, the teachers gave an estimation of LPE based upon their insights in the background of their students and involved three categories (1 = low educated parents, 2 = moderately educated parents, 3 = highly educated parents).

#### Self-Perceived Executive Functions: The Amsterdam Executive Function Inventory

The Amsterdam Executive Functioning Inventory (AEFI) was developed to measure executive aspects of daily life behavior by means of a self-report questionnaire (see van der Elst et al., 2012b for construction and psychometric details and Baars et al., 2015; van Tetering et al., 2018, 2020 for findings done with the AEFI). The inventory consists of 13 items that represent three dimensions of executive functioning: (1) Attention (three items); (2) Planning and initiative taking (five items); and (3) Self-control and self-monitoring (five items). The self-report version of the AEFI takes between 5 and 10 min to complete. The participants were asked to indicate how well each item described their ability on a three-point Likert scale: “1 = not true,” “2 = partly true,” “3 = true.” Items 1, 4, 5, 6, 7, 8, 11, 12, and 13 were reverse coded. The total score of all items was calculated, with higher scores indicating better EFs.

The construct validity and reliability of the AEFI were previously evaluated in a large study of adolescents aged 15–18 and have been reported to be adequate (van der Elst et al., 2012b). Internal consistency of the total AEFI has been re-evaluated in the samples of study 1 (Cronbach’s alpha = 0.705) and study 2 (Cronbach’s alpha = 0.667), and can be considered acceptable (i.e., alpha ≥ 0.6–0.7, Holden et al., 1991; Clark and Watson, 1995).

#### Underachievement and Behavioral Problems

In study 2, teachers were asked to judge each of their students about potential underachievement and behavioral problems on a 5-point scale (1 = totally true to 5 = totally not true). Underachievement was assessed using the following statement: “*This student can probably perform (much) better than is indicated by his/her grades. He/she is an underachiever.”* Behavioral problems were assessed by the following statement: “*This student is disturbing in class and shows behavioral problems (disinhibited, easily angry/aggressive, very busy, etc.).”*

### Statistical Analyses

In both studies, before testing regression models, Spearman’s rho correlations were calculated in study 1 and study 2, to examine the basic pattern of relationships between self-perceived EFs, LPE, sex, and school performance (CITO Mathematics and CITO Reading Comprehension and in study 2 also the CITO Final Achievement Test). As a second step, ordinal regression analyses were performed on the data in study 1, and hierarchical linear regression analyses were performed on the data in study 2. In both studies, CITO Mathematics and CITO Reading Comprehension were the dependent variables. In study 2, the CITO Total Achievement Test was another dependent variable. Sex, LPE and self-perceived EFs were entered as independent variables in study 1. In study 2, Sex and LPE were the independent variables in model 1, and self-perceived EFs was added as independent variable in model 2.

Additional analyses were performed in study 2 to investigate the relation between self-perceived EFs and school performance in underachievers and in students with behavioral problems. First, Spearman’s rho correlations were calculated to examine the basic pattern of relationships between self-perceived EFs, LPE, sex, and the dependent variables underachievement and behavioral problems. Then, ordinal regression analyses were performed with underachievement and behavioral problems as dependent variables. Sex, LPE, and self-perceived EFs were entered as independent variables. For underachievement, CITO Final Achievement Test scores was added as well.

The analyses in study 1 were performed in R, and the analysis in study 2 were performed in SPSS version 27.

## Results

### Demographics and Descriptive Statistics of Study 1

Demographics and the descriptive statistics of the population in study 1 are described in Table 1.

**TABLE 1:** Demographics and descriptive statistics of study 1.

	*N*	Mean	*SD*	Range (min-max score)/Frequencies (*N*)
Sex (% girls)	229	57.3%	−	−
LPE	169	−	−	Low: 31 Moderate: 91 High: 47
CITO mathematics	154	2.55	1.32	1–5
CITO reading comprehension	153	2.61	1.42	1–5
Self-perceived EFs	217	29.36	4.25	16–39

*N is the number of participants with complete data on the variable, and the LPE levels are explained in Supplementary Table 1.*

For study 1, intercorrelations among the CITO Mathematics and CITO Reading Comprehension (dependent variables) and the independent variables (sex, LPE, and self-perceived EFs) ranged from *r* = −0.03 to *r* = 0.57, as is shown in Table 2. Table 2 also illustrates that self-perceived EFs was significantly correlated with CITO Mathematics and CITO Reading Comprehension. Moreover, LPE was significantly correlated with CITO Mathematics and CITO Reading Comprehension, but there were no significant correlations with sex.

**TABLE 2:** Spearman’s rho correlations between the dependent and independent variables in study 1.

	2.	3.	4.	5.
Self-perceived EFs *N*	−0.29*** 144	−0.30*** 143	0.10 *162*	−0.03 *217*
CITO mathematics *N*		0.57*** 153	−0.23** 135	0.10 *154*
CITO reading comprehension *N*			−0.35*** 134	−0.03 *153*
LPE *N*				0.04 *169*
Sex (0 = boy, 1 = girl)				

Lower achievement levels on the CITO Mathematics and CITO Reading Comprehension tests indicate better performance; **p ≤ 0.01, ***p ≤ 0.001.

#### Relation Between Self-Perceived Executive Functions and Mathematics in Study 1

Table 3 presents results of the ordinal regression model with CITO Mathematics as dependent variable. A higher Self-Perceived EFs score and higher LPE had a significant negative impact on the CITO Mathematics score (with lower scores being indicative of better performance) (*p* ≤ 0.01). Sex had no impact on the Mathematic outcome (*p* > 0.05).

**TABLE 3:** Results of the ordinal regression analyses on the relation between self-perceived EFs and mathematics in study 1 (*N* = 129).

	β	SE	*t*-value
LPE_Moderate	–0.74	0.43	–1.74
LPE_High	–1.31	0.48	−2.73**
Sex	0.54	0.33	1.65
Self-perceived EFs	–0.14	0.04	−3.35***

Lower achievement levels on the CITO Mathematics test indicates better performance; **p ≤ 0.01, ***p ≤ 0.001.

With respect to LPE, CITO Mathematics score is significantly different between the lowest and highest LPE groups, but not between the lowest and moderate LPE groups. Compared to the lowest LPE group, CITO Mathematics score is significantly higher in the highest LPE group. There was no significant effect of sex.

#### Relation Between Self-Perceived Executive Functions and Reading Comprehension in Study 1

Table 4 presents results of the ordinal regression model with CITO Reading Comprehension as dependent variable. A higher Self-Perceived EFs score and higher LPE were associated with a significantly lower CITO Reading Comprehension score (with lower scores being indicative of better performance) (*p* ≤ 0.01). Sex had no impact on the Reading Comprehension outcome (*p* > 0.05).

**TABLE 4:** Results of the ordinal regression analyses on the relation between self-perceived EFs and reading comprehension in study 1 (*N* = 128).

	β	SE	*t*-value
LPE_Moderate	−0.82	0.44	–1.87
LPE_High	−1.84	0.50	−3.69***
Sex	−0.12	0.32	–0.37
Self-perceived EFs	−0.11	0.04	−2.72**

Lower achievement levels on the CITO Reading Comprehension test indicates better performance; **p ≤ 0.01, ***p ≤ 0.001.

With respect to LPE, CITO Reading comprehension score is significantly different between the lowest and highest LPE groups, but not between the lowest and moderate LPE groups. Compared to the lowest LPE group, CITO Reading Comprehension score is significantly higher in the highest LPE group. There was no significant effect of sex.

### Demographics and Descriptive Statistics of Study 2

Table 5 describes the demographics and descriptive statistics of the sample of study 2.

**TABLE 5:** Demographics and descriptive statistics of study 2.

	*N*	Mean	SD	Range (min-max score)/Frequencies (*n*)
Sex (% girls)	237	50.2%	−	−
LPE	217	−	−	1 (Low): 23 2 (Moderate): 121 3 (High): 73
CITO final achievement test	184	536.24	9.53	504–550
CITO mathematics	157	115.03	14.46	13–168
CITO reading comprehension	191	62.20	18.23	22–119
Self-perceived EFs	214	29.36	4.02	18–37

*N is the number of participants with complete data on the variable.*

Intercorrelations among the dependent variables (i.e., CITO Final Achievement Test, CITO Mathematics, and CITO Reading Comprehension) and the independent variables (i.e., sex, LPE, and self-perceived EFs) ranged from *r* = −0.01 to *r* = 0.80, as is shown in Table 6. Table 6 also illustrates that self-perceived EFs was significantly correlated with CITO Final Achievement Test, CITO Mathematics, CITO Reading Comprehension and LPE. Moreover, the CITO tests were all significantly correlated with LPE. As in Study 1, there were no significant correlations with sex.

**TABLE 6:** Spearman’s rho correlations between the dependent and independent variables in study 2.

	2.	3.	4.	5.	6.
Self-perceived EFs *N*	0.28*** 175	0.25** 147	0.17* 179	0.24*** 195	0.04 214
CITO final achievement test *N*		0.80*** 149	0.75*** 181	0.54*** 168	−0.01 184
CITO mathematics *N*			0.60*** 157	0.43*** 139	−0.01 157
CITO reading comprehension *N*				0.43*** 173	0.03 191
LPE *N*					−0.13 215
Sex (0 = boy, 1 = girl) *N*					

Higher skill scores on the CITO Mathematics and CITO Reading Comprehension tests and higher achievement on the CITO Final Achievement Test indicate better performance; *p ≤ 0.05, **p ≤ 0.01, ***p ≤ 0.001.

#### Relation between Self-Perceived Executive Functions and Mathematics in Study 2

Table 7 presents results of the hierarchical linear regression model with CITO Mathematics as dependent variable. Model 1, with sex and LPE as independent variables, was significant [*F*_(3, 135)_ = 3.76, *p* = 0.01]. In this model, CITO Mathematics and Higher LPE were significantly associated. This model accounted for 8% of the total variance in Mathematics.

**TABLE 7:** Results of the hierarchical linear regression analyses on the relation between self-perceived EFs and mathematical performance in study 2 (*N* = 139).

	B	*t*	*R* ^2^	Δ*R*^2^	Δ*F*
**Model 1**			0.08	0.06	3.76**
Constant	111.42	3.92***			
Sex	0.57	2.42			
LPE_Moderate	0.50	4.01			
LPE_High	8.99	4.23*			
**Model 2**			0.09	0.06	3.22*
Constant	100.37	9.72***			
Sex	0.35	2.43			
LPE_Moderate	0.66	4.01			
LPE_High	8.23	4.26*			
Self-perceived EFs	0.39	0.31			

Higher skill scores on the CITO Mathematics test indicate better performance; *p ≤ 0.05, **p ≤ 0.01, ***p ≤ 0.001.

When self-perceived EFs was added into model 2, the model was still significant [*F*_(4, 134)_ = 3.22, *p* = 0.02], but the amount of variance did not significantly increase (Δ*R*^2^ = 0.06). In this model, only Higher LPE was significantly related to CITO Mathematics. The relation between self-perceived EFs and CITO Mathematics did not reach significance.

#### Relation Between Self-Perceived Executive Functions and Reading Comprehension in Study 2

Table 8 presents results of the hierarchical linear regression model with Reading Comprehension as dependent variable. Model 1, with sex and LPE as independent variables, was significant [*F*_(3_, _169)_ = 11.05, *p* < 0.001]. In this model, CITO Reading Comprehension and Moderate and High LPE were significantly associated. This model accounted for 16.4% of the total variance in CITO Reading Comprehension.

**TABLE 8:** Results of the hierarchical linear regression analyses on the relation between self-perceived EFs and reading comprehension performance in study 2 (*N* = 173).

	B	*t*	*R* ^2^	Δ*R*^2^	Δ*F*
**Model 1**			0.16	0.15	11.05***
Constant	46.71	4.18***			
Sex	3.70	2.58			
LPE_Moderate	10.59	4.27**			
LPE_High	22.90	4.50***			
**Model 2**			0.17	0.15	8.74
Constant	34.47	10.36***			
Sex	3.46	2.58			
LPE_Moderate	10.76	4.27**			
LPE_High	22.06	4.54***			
Self-perceived EFs	0.43	0.33			

Higher skill scores on the CITO Reading test indicate better performance; **p ≤ 0.01, ***p ≤ 0.001.

When self-perceived EFs was added into model 2, the model was still significant [*F*_(4, 168)_ = 8.74, *p* < 0.001], but the amount of variance did not significantly increase (Δ*R*^2^ = 0.15). In this model, Moderate and High LPE were related to CITO Reading Comprehension performance. The relation between self-perceived EFs and CITO Reading Comprehension did not reach significance.

#### Relation Between Self-Percieved Executive Functions and CITO Final Achievement Test

Table 9 presents results of the hierarchical linear regression model with CITO Final Achievement Test as dependent variable. Model 1, with sex and LPE as independent variables, was significant [*F*_(3, 164)_ = 17.84, *p* < 0.001]. In this model, CITO Final Achievement Test was significantly associated with Moderate and High LPE. This model accounted for 24.6% of the total variance in CITO Final Achievement Test.

**TABLE 9:** Results of the hierarchical linear regression analyses on the relation between self-perceived EFs and CITO final achievement test in study 2 (*N* = 168).

	B	*t*	*R* ^2^	Δ*R*^2^	Δ*F*
**Model 1**			0.25	0.23	17.84***
Constant	526.39	2.10***			
Sex	0.94	1.30			
LPE_Moderate	7.64	2.15***			
LPE_High	15.19	2.27***			
**Model 2**			0.27	0.26	15.27***
Constant	514.89	5.15***			
Sex	0.70	1.29			
LPE_Moderate	7.80	2.12***			
LPE_High	14.40	2.26***			
Self-perceived EFs	0.40	0.17**			

Higher achievement on the CITO Final Achievement Test indicates better performance; **p ≤ 0.01, ***p ≤ 0.001.

When self-perceived EFs was added into model 2, the model was still significant [*F*_(4, 163)_ = 15.27, *p* < 0.001] and the amount of variance explained increased to 27.3% (Δ*R*^2^ = 0.26). In this model, Moderate and High LPE, and self-perceived EFs were significantly related to CITO Final Achievement Test.

### Secondary Analyses: Underachievers and Students With Behavioral Problems in Study 2

Teachers evaluated the Underachievement (*M* = 3.8, SD = 1.05, range: 1–5) and the Behavioral Problems (*M* = 4.4, SD = 0.98, range 1–5) of *N* = 160 students. Intercorrelations among Underachievement, Behavioral Problems (dependent variables) and the independent variables (sex, LPE and self-perceived EFs) ranged from *r* = −0.01 to *r* = 0.54, as is shown in Table 10. Table 10 also illustrates that self-perceived EFs was significantly correlated with LPE, Underachievement, and Behavioral Problems.

**TABLE 10:** Spearman’s rho correlations between the dependent and independent variables in underachievers and children with behavioral problems in study 2.

	2.	3.	4.	5.	6.
Self-perceived EFs *N*	0.25*** 198	0.32*** 198	0.28*** 175	0.24** 195	0.04 *214*
Underachievement *N*		0.28*** 218	0.24* 171	0.27*** 215	−0.08 *218*
Behavioral problems *N*			0.20** 171	0.08 *215*	0.22** 218
CITO final achievement test *N*				0.54*** 168	−0.01 *184*
LPE *N*					0.07 *215*
Sex (0 = boy, 1 = girl) *N*					

Underachievement and behavioral problems were scored on a 5-point scale with 1 = totally true and 5 = totally not true; higher achievement on the CITO Final Achievement Test indicates better performance; *p ≤ 0.05, **p ≤ 0.01, ***p ≤ 0.001.

#### Underachievers

Table 11 presents results of the ordinal regression model with Underachievement as dependent variable. Higher CITO Final Achievement Test performance (*p* ≤ 0.001) and moderate LPE (*p* < 0.01) were associated with a, respectively, positive (higher CITO performance was associated with higher self-perceived EFs) and negative (compared to lower LPE, Moderate LPE was associated to lower score on Underachievement) significant impact on Underachievement (*p* < 0.01). Sex and self-perceived EFs had no impact on the Underachievement outcome (*p* > 0.05).

**TABLE 11:** Results of the ordinal regression analyses on the relation between self-perceived EFs and underachievement in study 2 (*N* = 160).

	β	SE	*t*-value
LPE_Moderate	–2.06	0.70	−3.0**
LPE_High	–0.78	0.73	–1.07
CITO final achievement test	0.03	0.003	12.71***
Sex	–0.39	0.30	–1.29
Self-perceived EFs	0.05	0.04	1.14

Underachievement was scored on a 5-point scale with 1 = totally true and 5 = totally not true; higher achievement on the CITO Final Achievement Test indicates better performance; **p ≤ 0.01, ***p ≤ 0.001.

#### Behavioral Problems

Table 12 presents results of the ordinal regression model with Behavioral problems as dependent variable. Sex and self-perceived EFs were associated with a positive impact on behavioral problems (girls and higher self-perceived EFs were associated with higher scores on self-perceived EFs, *p* < 0.05). LPE had no impact on the Behavioral Problems outcome (*p* > 0.05).

**TABLE 12:** Results of the ordinal regression analyses on the relation between self-perceived EFs and classroom behavioral problems in study 2 (*N* = 195).

	β	SE	*t*-value
LPE_Moderate	–0.35	0.68	–0.51
LPE_High	0.17	0.72	0.24
Sex	0.81	0.34	2.38*
Self-perceived EFs	0.14	0.04	3.10**

Behavioral problems were scored on a 5-point scale with 1 = totally true and 5 = totally not true; *p ≤ 0.05, **p ≤ 0.01.

## Discussion

The primary aim of this study was to evaluate the relation between the self-perceived EFs of young adolescents and school performance, while controlling for sex and LPE. This was investigated in two separate studies each involving more than 200 subjects in 20 mainstream primary schools (12 schools in study 1 and 8 schools in study 2). In both studies, the results indicated that the self-perceived EFs of young adolescents were related to their school performance. In addition, school achievement and self-perceived EFs were both related to LPE. Sex did not play a statistically significant role. Furthermore, besides the relation between self-perceived EFs and school results, we also found a relation between self-perceived EFs and teacher ratings of behavioral problems in the classroom.

### Executive Functions and School Performance

Results revealed a positive correlation between self-perceived EFs and school performance of young adolescents in both studies. The higher the young students evaluated their EFs, the higher their mathematics and reading comprehension performance. In the second study, the self-evaluated EFs of young students were also positively correlated to a more robust measure of school performance: the CITO Final Achievement Test. This measure is a standardized tool to measure language and arithmetic-mathematics abilities at the end of sixth grade. It is used annually by most Dutch primary schools. After sixth grade, young students in the Netherlands make the transition from primary to secondary education. Performance on the CITO Final Achievement Test aids in choosing the level of secondary education that matches Students’ cognitive abilities. Our results show that there is a positive correlation between Students’ EF self-evaluations and CITO performance: the higher young students evaluate their EFs at the end of primary school, the higher their performance on this test. It is not yet clear to what extent Student’s EFs directly or indirectly play a role in selecting secondary education, but this is a question worth exploring in the future.

Our findings confirm and extend prior research, which shows a relationship between EFs and academic achievement in various age groups using other methodologies including cognitive tasks and observer-reports (Kent et al., 2014; Baars et al., 2015; Dekker et al., 2017; Nije Bijvank et al., 2017; van Tetering and Jolles, 2017). This finding is important as self-reports do not necessarily show a large correspondence with task-based assessments of cognitive functioning (e.g., Fine et al., 2016). Therefore, our findings suggest that besides the core EFs, executive aspects of daily life functioning may be relevant predictors of academic achievement as well. Although we cannot rule out the influence of a third variable affecting both self-reported EFs and academic test performance, it is not unlikely that Students’ EFs have a general and direct effect on learning and/or academic test performance. For example, prior research suggests that young students with lower levels of EFs have more difficulties to sustain attention while performing a test (Anderson, 2002; Diamond, 2013). Moreover, students with lower EFs may also have difficulties with planning and prioritizing the steps needed to solve an assignment, and they have difficulties in suppressing irrelevant impulses and information while taking a test (Diamond, 2013; Gerst et al., 2015). Poorer performance on the higher-order EFs could thus directly and negatively affect test performance. In addition, better attention, improved self-control, and adequate planning skills may be beneficial for learning. They may support the students to identify relationships both within new topics and themes for learning, and between different subjects. In addition, students with stronger skills may be better able to adjust their behavior toward new situations (Anderson, 2002; Gerst et al., 2015; Jolles, 2016; Zelazo et al., 2016). Furthermore, EFs may contribute to the social monitoring skills, which allow students to grasp the intentions of others and often play an important role in extracting key information from a learning situation (Jolles, 2016). As a result, children and young teens with stronger EFs have an advantage that could help them to acquire more knowledge and to obtain higher educational levels later in life. This has also been suggested by other researchers and in our earlier papers (e.g., Best et al., 2011; Diamond, 2013; Friso-van den Bos et al., 2013; Arrington et al., 2014; Cragg and Gilmore, 2014; Gerst et al., 2015; Dekker et al., 2017; van Tetering and Jolles, 2017; van Tetering et al., 2018).

### Executive Function Self-Evaluation in Early Adolescence

Our results suggest that at the age of 10–12 years, young adolescents can evaluate their EFs in a way that is relevant to their school achievement. The relationship between self-evaluated EFs and school performance is consistent with and extends our prior findings (van Tetering et al., 2018). In our prior work, teachers evaluated the EFs of young students on a teacher-version of the questionnaire that was used in the present study (i.e., the AEFI). Students who received higher evaluations by their teachers performed better in school compared to students who received lower evaluations. This suggests that teacher-evaluations provide valuable information about non-academic functioning of young adolescents in school. Building on these findings, our present results give an important indication about EF skills: young adolescents aged 9–12—generally—can reflect on their own functioning, including their ability to organize schoolwork, to sustain attention, and to ignore distracting situations. As these abilities are important for learning in school, the finding that young students can properly reflect on them may have important implications for further development of these abilities in the academic context. In addition, our findings may be of practical value for research into EF development, revealing the potential of using self-reports to get insight into Students’ EFs in daily life. The use of self-reports—as opposed to teacher-reports—is particularly relevant for the age period of adolescence as the intensity of supervision by teachers gradually declines across adolescence. Teachers no longer observe all scholastic activities of their students and they have even less insight into daily life activities outside of the school environment, while these school-extrinsic activities may impact homework assignments and study motivation. Accordingly, teachers may lose sight on possible weaknesses in Students’ EFs that could have negative consequences for their school performance. A major finding of our study is thus that the self-reports of young adolescents can be used to gain information on their EFs in these day-to-day and school situations. An important direction for future research is to examine consistencies and differences between self-reports and observer reports in adolescents, and to investigate the extent to which a composite measure of self-report and observer reports provides a more robust predictor of daily life behavior than either measure alone.

### The Role of Parental Education and Male/Female Sex

Prior work suggests that family background plays an important role in the relation between EFs and school achievement (Hurks et al., 2006; Meijs et al., 2009; Rindermann and Baumeister, 2015; van Tetering et al., 2018). In agreement with these findings, our results showed that LPE was associated with both school performance and self-evaluated EFs of young adolescents, although the relation between LPE and self-evaluated EFs was only significant in sample 2. It has been demonstrated that well-educated parents tend to create a more intellectually stimulating environment for their children than parents with lower education (e.g., Hoff, 2003; Rindermann and Baumeister, 2015). A stimulating environment affects the complexity of language used, the number and quality of the books read, the availability of playing materials, the level of ambitions that parents have for their developing child, as well as school attendance and general cognitive development (Ganzach, 2000; Hoff et al., 2002; Carr and Pike, 2012; Kautz et al., 2014). It is not unlikely that these factors have a positive influence on the development of EFs and contribute to better school achievement. This has a direct implication that children from less educated families could profit from support from educators outside the home situation to stimulate them in their development of EFs.

With respect to sex differences, we did not find a significant difference between self-perceived EFs of boys and girls in the present study, nor was there an effect of sex on school performance. This finding is of interest because it is not in line with some earlier studies assessing EFs with other methods (behavioral tasks or observer reports) in this age-group. For instance, behavioral studies showed that girls outperformed boys on a verbal fluency task (Barel and Tzischinsky, 2018) and on an information processing speed task (van der Elst et al., 2012a; Dekker et al., 2013). Moreover, it has been demonstrated that boys have lower levels of inhibitory control than girls (Loyer Carbonneau et al., 2020). In addition to these sex differences on behavioral tasks, in one of our earlier studies teachers reported boy-girl differences in self-control and self-monitoring skills of 9–12-year-olds (van Tetering and Jolles, 2017). Thus, although sex differences were established on EF tasks and by observer reports, the present findings suggest that young adolescents do not notice these differences themselves. This is in line with a prior study using self-reports, which showed sex differences in the self-perceived EFs of 13–16-year-olds, but not in the evaluations of 10–12-year-olds (van Tetering et al., 2020). A potential explanation for the discrepancy between self-report and other types of measures is that students may reflect on their abilities by comparing themselves with their peers of the same sex.

Importantly, in study 1, the relation between self-perceived EFs and school achievement remained significant after controlling for LPE and sex. This finding is in line with findings from a large-scale cohort study including 1,265 children, which showed that the relation between childhood self-control and future academic outcomes (i.e., gaining a university degree) remains significant when controlling for gender, IQ and SES (e.g., Fergusson et al., 2013). In study 2, self-perceived EFs also explained additional variance in performance on the CITO Final Achievement Test scores, over and above LPE and sex. Yet, self-perceived EFs did *not* contribute significantly to math and reading comprehension in this study after controlling for LPE. This suggests that in study 2, the relationship between EFs and math and reading achievement was primarily driven by differences in LPE. It should be noted that LPE was operationalized differently in both studies, which may have influenced our results. The LPE can be considered more accurate in study 1, where parents rated their highest level of education obtained on an internationally used classification system (Singh, 2010), compared to study 2, where teachers rated the LPE of each student on a 3-point scale (i.e., low, middle or high education). Yet, another possible explanation for the discrepancy between study 1 and study 2 on this finding is a difference in the average and variability of LPE between the two study samples, as the sample in study 1 has a higher average and smaller variability in LPE than the sample in study 2. This may have resulted in a smaller effect of LPE, and a greater *unique* contribution of EFs to school performance. In line with this idea, a recent meta-analysis demonstrated that samples with smaller SES variability showed significantly smaller correlations between SES and EF skills than samples with higher SES variability (Lawson et al., 2018).

Together, these and our findings imply that it is important to take the socio-economic background of a study population into consideration when investigating the influence of LPE on the relation between cognitive abilities and academic performance. Interestingly, a recent study has shown that the relationship between SES (including LPE), EFs, and academic achievement may also vary between different countries, suggesting that the effect of SES is not culturally universal (Ellefson et al., 2020). Taken together, for future research, it appears that the factor LPE deserves special attention when investigating the relation between EFs and school performance.

### Executive Functions Are Related to Behavioral Problems in the Classroom

In addition to our primary research questions, study 2 addressed two follow-up questions regarding the relationship between self-reported EFs on the one hand and teacher ratings of underachievement and behavioral problems on the other. Underachievers were defined as children who are characterized by lower school performance than would be expected based on their intellectual abilities. Our results showed that students who reported lower EFs were not significantly more likely to be judged an underachiever by their teacher, when controlling for absolute levels of school performance, LPE, and sex. This non-significant effect might be caused by the fact that we used a relatively rough and subjective measure of underachievement, consisting of only one item. Interestingly, we did find a significant relation between lower self-reported EFs and behavioral problems in the classroom as indicated by the teacher when controlling for sex and LPE. These findings are in line with and extend prior studies that revealed a relation between poor self-control and externalizing behavior or delinquency (e.g., Fergusson et al., 2013; Fine et al., 2016). Our finding shows that EFs are also relevant to consider when looking at less severe measures of disrupting behavior. Nevertheless, as this effect relies on one item, our findings warrant replication in future research with more comprehensive measures. As students who are disturbing in the classroom are at risk for not developing their full cognitive potential, investigation and stimulating the higher-order EFs might be a way to enhance school achievement (e.g., Ursache et al., 2012). As it appears that these early adolescents can evaluate major aspects of their executive functioning in daily and scholastic situations, it may be valuable to intervene in EF development in this important age period.

## Concluding Remarks

In the lower grades and especially elementary school, teachers and parents often help students to stay focused, plan and organize, especially those who have weaker self-control. This support wanes as students get older and in the developmental process from preadolescence to early and middle adolescence (e.g., Steinberg, 2014; Jolles, 2016), because children and teens gain experience and become more independent (Steinberg, 2014). By the end of primary school, students are expected to plan their homework, understand the intentions of the teacher, and adjust their behavior to the requirements of the school environment. Secondary education demands even more independence, initiative, and self-reliance, especially when students are expected to work by themselves on assignments that take a longer time to complete. Yet, prior research has indicated that EFs continue to develop across adolescence (e.g., Diamond, 2013). Furthermore, it has become increasingly clear that there are differences between individuals in the strength of their EFs, which appear to be relevant for school performance. This applies to the core EFs, but also to executive aspects of daily life behavior, as was demonstrated in the current study. Therefore, rather than focusing on academic content, education should also aim to stimulate the neuropsychological development of students and help in the acquisition of the skills that are essential for learning (Zelazo et al., 2016). Accordingly, schools could benefit from including activities in their curricula that stimulate the development of Students’ EFs. This is of special importance for children from lower educated families who may lag behind in the development of EFs because their home environment has not been optimal for the acquisition of skills in this domain (Rindermann and Baumeister, 2015; Khundrakpam et al., 2019; Mackes et al., 2020). Moreover, results from the present study indicate that not only children from lower LPE families may benefit from such facilities, but also students with behavioral problems in the classroom.

## Data Availability Statement

The datasets presented in this study can be found in an online repository: doi: 10.34894/YDXMLQ. Requests to access the dataset can be made to DJ via the repository page.

## Ethics Statement

The studies involving human participants were reviewed and approved by the Ethics Committee of the Faculty of Educational Sciences of the Leiden University and the Ethical Committee of the Faculty of Behavioral and Movement Sciences of the Vrije Universiteit Amsterdam. Written informed consent to participate in this study was provided by the participants’ legal guardian/next of kin and the participants themselves.

## Author Contributions

MvT: substantial contributions to the conception and design of the work, the analysis, and interpretation of data for the work, drafting the work. WE: the analyses and interpretation of data for the work, revising the analytical work critically. DJ and JJ: substantial contributions to the conception or design of the work, the acquisition and interpretation of data for the work, revising the work critically for important intellectual content. All authors contributed to the article and approved the submitted version and agree to be accountable for all aspects of the work in ensuring that questions related to the accuracy or integrity of any part of the work are appropriately investigated and resolved.

## Conflict of Interest

WE was employed by company Johnson & Johnson. The remaining authors declare that the research was conducted in the absence of any commercial or financial relationships that could be construed as a potential conflict of interest.

## Publisher’s Note

All claims expressed in this article are solely those of the authors and do not necessarily represent those of their affiliated organizations, or those of the publisher, the editors and the reviewers. Any product that may be evaluated in this article, or claim that may be made by its manufacturer, is not guaranteed or endorsed by the publisher.
