# Supplementary material for: School Achievement in Early Adolescence Is Associated With Students’ Self-Perceived Executive Functions
Source: Front Psychol. 2022 Mar 14;12:734576. doi: 10.3389/fpsyg.2021.734576 (PMC8964458; doi:10.3389/fpsyg.2021.734576)
Supplement: Supplementary file 1 [file Table_1.DOCX]

Supplementary Material

# Supplementary Data

*Supplementary Table 1. LPE levels of study 1.*

| **Level** | **Specification** |
| --- | --- |
| 0 | Lower education |
| 1 | Lower professional education (LBO, LTS, LEAO, LHNO, huishoud- of ambachtsschool, VMBO-kader of basis*) |
| 2 | Middle education (ULO, MULO, MAVO, VMBO-T*) |
| 3 | Middle professional education (MBO, MTS, MEAO*) |
| 4 | Higher education and higher secondary school (HAVO, HBS, MMS, gymnasium, Lyceum, VWO*) |
| 5 | Higher professional education (HBO*) |
| 6 | University |

**For more information on the abbreviations see:* [*https://en.wikipedia.org/wiki/Education_in_the_Netherlands*](https://en.wikipedia.org/wiki/Education_in_the_Netherlands)*.*

**
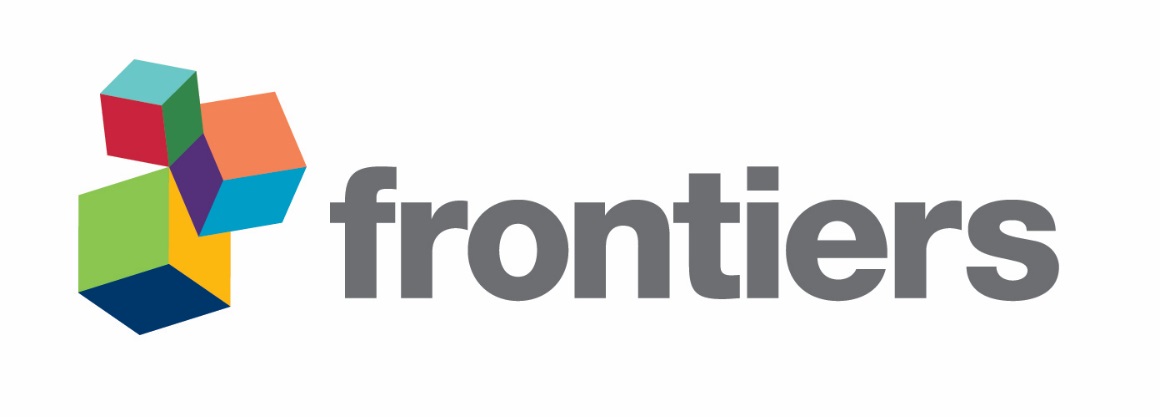
**
